# A new transgene mouse model using an extravesicular EGFP tag enables affinity isolation of cell-specific extracellular vesicles

**DOI:** 10.1038/s41598-021-04512-0

**Published:** 2022-01-11

**Authors:** Mikkel Ø. Nørgård, Lasse B. Steffensen, Didde R. Hansen, Ernst-Martin Füchtbauer, Morten B. Engelund, Henrik Dimke, Ditte C. Andersen, Per Svenningsen

**Affiliations:** 1 Department of Molecular Medicine, Cardiovascular and Renal Research, University of Southern Denmark, J.B. Winsloews vej 21.3, 5000 Odense C, Denmark; 2 Department of Molecular Biology and Genetics, Aarhus University, Aarhus C, Denmark; 3 Department of Clinical Genetics, Odense University Hospital, Odense C, Denmark; 4 Clinical Genome Center, University of Southern Denmark, Odense C, Denmark; 5 Department of Nephrology, Odense University Hospital, Odense C, Denmark; 6 DCA-Lab, Department of Clinical Biochemistry and Pharmacology, Odense University Hospital, Odense C, Denmark; 7 Department of Clinical Research, University of Southern Denmark, Odense C, Denmark

**Keywords:** Cell biology, Molecular biology, Biomarkers

## Abstract

The in vivo function of cell-derived extracellular vesicles (EVs) is challenging to establish since cell-specific EVs are difficult to isolate and differentiate. We, therefore, created an EV reporter using truncated CD9 to display enhanced green fluorescent protein (EGFP) on the EV surface. CD9truc-EGFP expression in cells did not affect EV size and concentration but enabled co-precipitation of EV markers TSG101 and ALIX from the cell-conditioned medium by anti-GFP immunoprecipitation. We then created a transgenic mouse where CD9truc-EGFP was inserted in the inverse orientation and double-floxed, ensuring irreversible Cre recombinase-dependent EV reporter expression. We crossed the EV reporter mice with mice expressing Cre ubiquitously (*CMV-Cre*), in cardiomyocytes (*αMHC-MerCreMer*) and renal tubular epithelial cells (*Pax8-Cre*), respectively. The CD9truc-EGFP positive mice showed Cre-dependent EGFP expression, and plasma CD9truc-EGFP EVs were immunoprecipitated only from CD9truc-EGFP positive *CD9truc-EGFPxCMV-Cre* and *CD9truc-EGFPxαMHC-Cre* mice, but not in *CD9truc-EGFPxPax8-*Cre and CD9truc-EGFP negative mice. In urine samples, CD9truc-EGFP EVs were detected by immunoprecipitation only in CD9truc-EGFP positive *CD9truc-EGFPxCMV-Cre* and *CD9truc-EGFPxPax8-Cre* mice, but not *CD9truc-EGFPxαMHC-Cre* and CD9truc-EGFP negative mice. In conclusion, our EV reporter mouse model enables Cre-dependent EV labeling, providing a new approach to studying cell-specific EVs in vivo and gaining a unique insight into their physiological and pathophysiological function.

## Introduction

Extracellular vesicles (EVs) are nanosized membrane-bound vesicles that may function as mediators of cell–cell communication by transfer of cellular proteins, lipids, and nucleic acids^1^. The presence of EVs in biological fluids, such as urine and plasma, has also gained clinical interest since it facilitates monitoring physiological and pathophysiological processes using minimally invasive techniques. While EVs potentially enable the non-invasive interrogation of cells buried deep within an organism, the cell-specific isolation and tracking of EVs in vivo remain challenging since EVs are secreted from almost every cell types^2–9^. This hampers our ability to investigate the role of EVs in a physiologically relevant context.

EVs are a heterogeneous group of cell-derived vesicles, and the two main forms are exosomes and microvesicles. Their biogenesis differs: microvesicles directly bud off the plasma membrane, while exosomes are of endosomal origin and accumulate in intraluminal vesicles (ILVs) in multivesicular bodies (MVBs). EVs are, however, enriched in tetraspanins, such as CD63 and CD9^10–12^, which are transmembrane proteins widely distributed in the plasma membrane^13^. The tetraspanins are considered valid markers of EVs^14^ and have been widely used for the isolation and tracking of EVs.

The tetraspanins have intracellular N- and C-terminals that enable genetic fusion of fluorescent reporter proteins and luciferase^15–19^. This strategy allows for tissue-specific signals exclusively from EVs and thereby overcome major limitations of previous approaches using radioisotopes, fluorescent dyes, and magnetic conjugated nanoparticles conjugated to lipophilic reagents to label EVs^7–9^. The lipophilic reagents may be released from the EVs, resulting in the distribution of non-EV-associated fluorescent signal^12,13^. Furthermore, infusing labeled EVs generated in vitro into mice may not be at physiologically relevant concentrations. While N and C terminal fusion of reporter proteins to the tetraspanins enables tracking of cell-specific EVs, these reporter proteins do not allow for affinity isolation of the labeled EVs from biological fluids in that the tetraspanin terminals are located inside EVs^12,13^.

Tetraspanins, however, can be used to display fluorescent proteins on the EV surface^17^, and we, therefore, hypothesized that the fusion of EGFP to the C-terminal of a truncated form of the tetraspanin CD9, devoid of the large extracellular domain and the last transmembrane domain, would create fluorescence-labeled EVs with affinity tags. Furthermore, flanking the inverted EV reporter with loxP sites^20^ would establish a genetic switch that enabled Cre recombinase-dependent EV reporter expression in vivo and allow easy and reliable tracking and isolation of cell-specific EVs. Using this novel transgenic mouse model enables the assimilation of new knowledge regarding EVs' physiological and pathophysiological functions.

## Results

### Fusion of EGFP to CD9 enable affinity isolation of EVs

**Figure 1:**
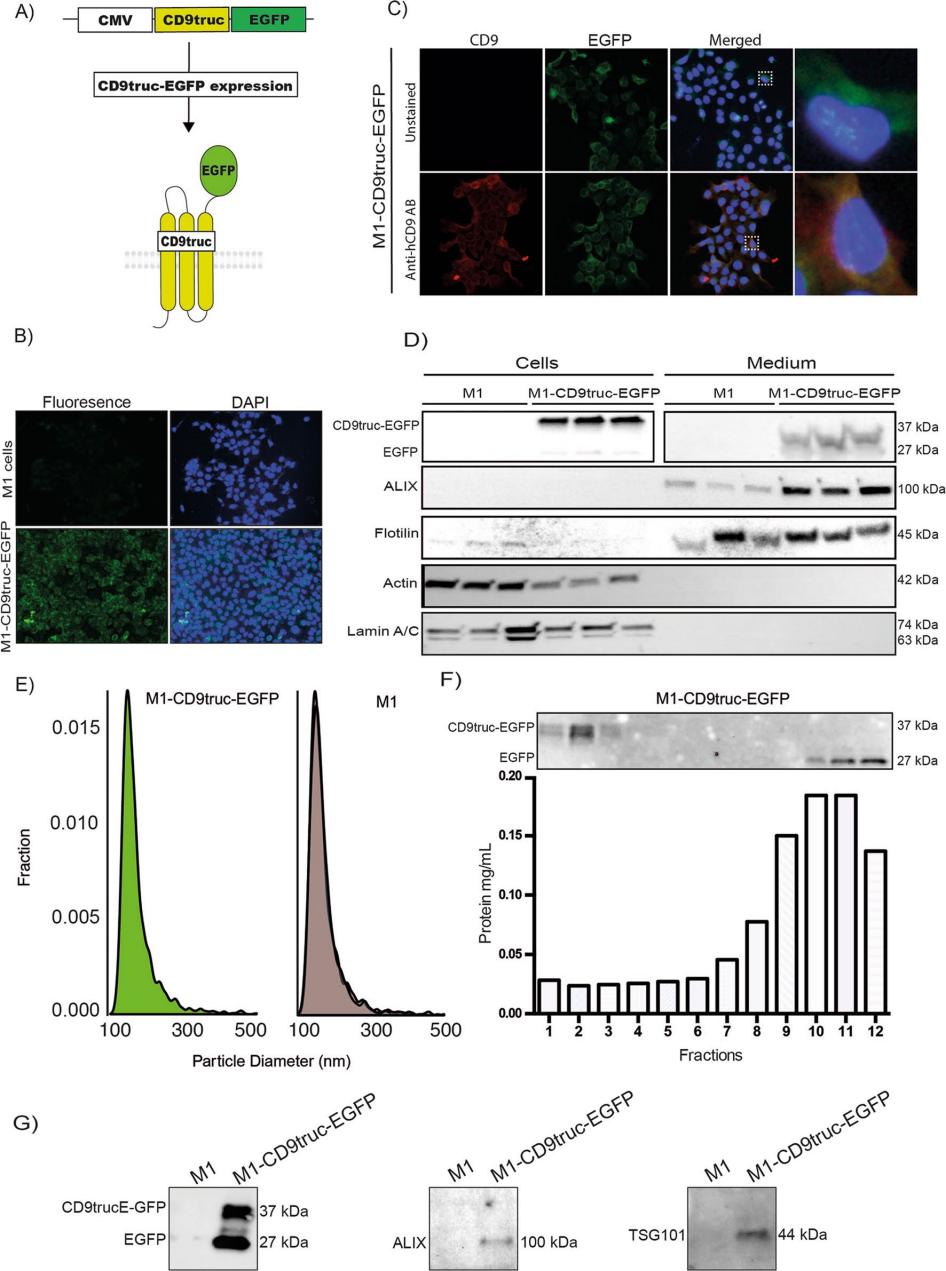
Expression of EGFP and associated EV markers M1-CD9truc-EGFP cells and their conditioned medium. (**A**) Illustration of our EV reporter gene and protein. The CD9truc-EGFP coding sequence is driven by the CMV promoter and encodes a fusion protein consisting of the first 3 transmembrane domains of CD9 and EGFP, enabling genetic labeling and EV surface display of EGFP. (**B**) M1 cells stable transfected with CD9truc-EGFP are green fluorescent in contrast to non-tansfected M1 cells. Nuclei (blue) and GFP (green). n = 3, ×200 magnification (**C**) Immunofluorescence labeling of CD9 in M1-CD9truc-EGFP cells using Anti-hCD9 AB shows colocalization between CD9 (red), EGFP (green) and nuclei (blue). ×200 magnification (n = 3) (**D**) M1 cells stable transfected with CD9truc-EGFP expressing a band reacting with an anti-GFP antibody in cells and PEG-precipitated conditioned medium. Actin and Lamin A/C were detected in cell lysates only, and EVs markers ALIX and Flotillin were detected in conditioned medium from transfected and non-transfected M1 cells (n = 3). Full-length blots are shown in Supplementary Figure S2A-F. (**E**) Tunable resistive pulse sensing on the conditioned medium from non-transfected M1 cells and stable transfected CD9truc-EGFP cells indicated that the size distribution of EVs is not affected the expression reporter proteins (n = 3). (**F**) Size-exclusion chromatography fractions of CD9truc-EGFP cell-conditioned medium showed full-length CD9truc-EGFP in fractions 1–3, while shedded EGFP was present in fractions 10–12 (n = 3). Full-length blots are shown in Supplementary Figure S2G. Below the western blot, the protein concentration in each fraction is shown (**G**) Anti-GFP immunoprecipitation of cells conditioned medium co-isolates CD9truc-EGFP and EV markers ALIX and TSG101 only in M1 cells stable transfected with CD9truc-EGFP (n = 3). Full-length blots are shown in Supplementary Figure S3A-C.

We designed an EV reporter protein by fusion of mouse CD9 truncated after the first 117 amino acids (the third transmembrane domain) to EGFP (Fig. 1A). The predicted molecular weight of the fusion protein is 41.5 kDa. Stably transfected epithelial M1 cells–termed M1-CD9truc-EGFP cells-showed green fluorescent intracellular vesicles co-localized with CD9 (Fig. 1B and C). Transfected fluorescent markers for the endoplasmic reticulum (mCherry-ER), early endosome marker Rab5 (mRFP-Rab5), and the tetraspanin CD81 (mTagBFP2-CD81) showed co-localization with EGFP in CD9truc-EGFP cells (Supplemental Figure S1A). Western blotting revealed EGFP expression restricted to transfected cells (Fig. 1D, the full-length blot is shown in Supplemental Figure S2A). While polyethylene glycol (PEG) precipitated cell-conditioned medium from M1 and M1-CD9truc-EGFP cells revealed enriched EV markers ALIX and flotillin, and not $\upbeta$-actin or nuclear Lamin A/C, EGFP was detected only in cell-conditioned medium from M1-CD9truc-EGFP cells (Fig. 1D, the full-length blots are shown in Supplemental Figure S2B-F). We noted that without the addition of protease inhibitors to the cell-conditioned medium, EGFP was present as two bands: one at ~ 37 kDa, slightly below the expected molecular weight of the fusion protein, and one at ~ 27 kDa, indicating partial proteolytic shedding of EGFP (Supplemental Figure S2B). Conditioned medium from M1 and M1-CD9truc-EGFP cells showed similar extracellular particle concentration and size distribution (Fig. 1E). Size-exclusion chromatography (SEC) of the cell-conditioned medium from M1-CD9truc-EGFP cells showed that full-length CD9truc-EGFP band was only present in the fractions 1–3 with a low protein content, while shedded EGFP was present in fractions 10–12 characterized by a higher protein content (Fig. 1F, the full-length blot is shown in Supplemental Figure S2G). This indicates that full-length CD9truc-EGFP is associated with EVs. Consistent with this, EGFP immunoprecipitation from cell-conditioned medium isolated EGFP and EV markers TSG101 and ALIX only from M1-CD9truc-EGFP cells (Fig. 1G, the full-length blot is shown in Supplemental Figure S3A-C). Thus, EVs from CD9truc-EGFP expressing cells can be isolated using GFP immunoprecipitation.

### Cre recombinase-dependent CD9truc-EGFP expression

**Figure 2:**
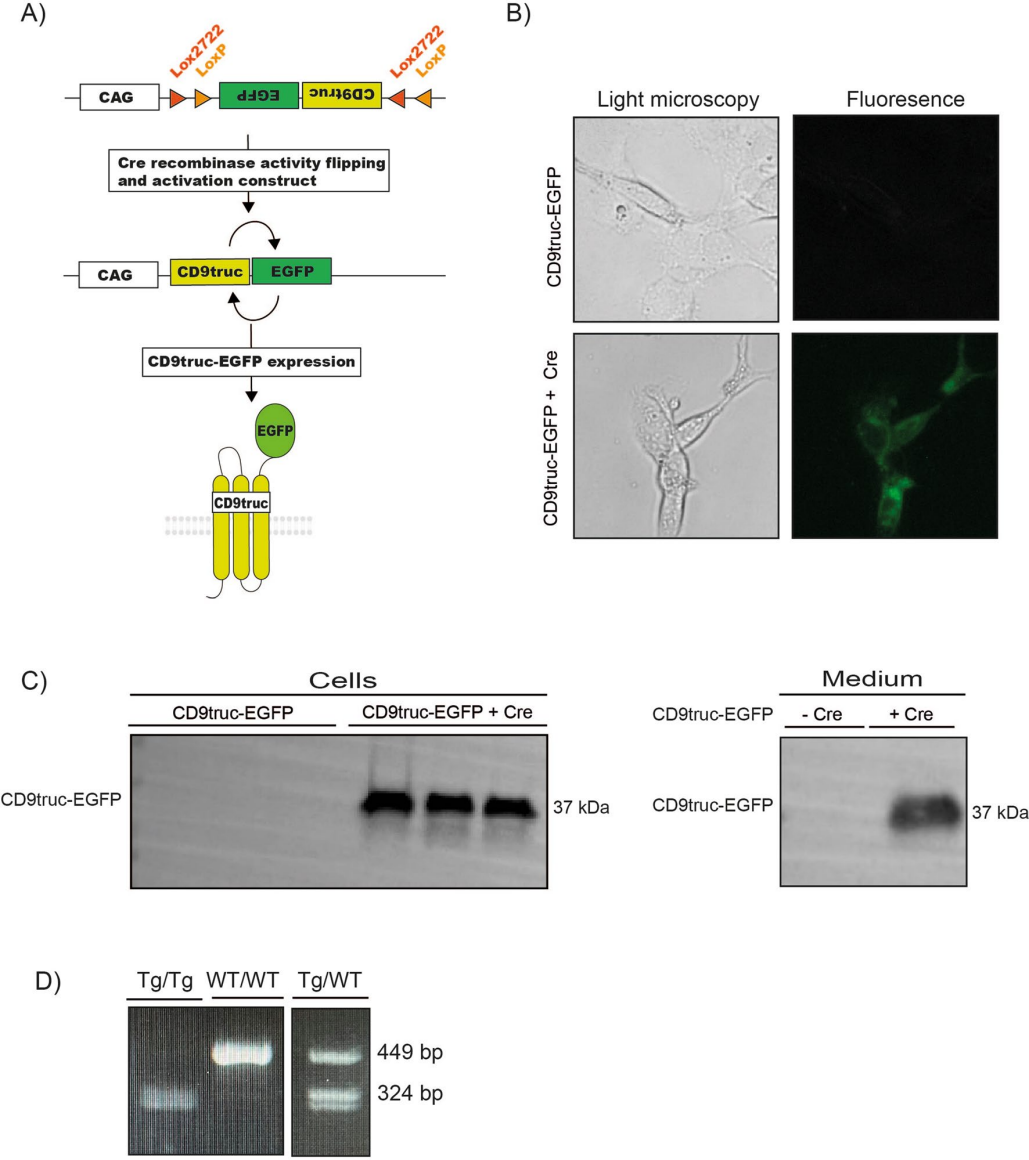
Cre recombinase-dependent expression of CD9truc-EGFP. (**A**) Our EV reporter *CD9truc-EGFP* was inverted and flanked by double lox sites. Upon Cre recombinase expression, the *CD9truc-EGFP* is inverted and yields CD9truc-EGFP expression driven by the CAG promoter. (**B**) HEK293T cells transiently transfected with double-floxed and inverted CD9truc-EGFP only express EGFP when co-transfected with Cre recombinase (n = 3). ×200 magnification (**C**) HEK293T cells co-transfected with double-floxed and inverted CD9truc-EGFP expressed CD9truc-EGFP in cells and conditioned medium (n = 3. Full-length blots are shown in Supplementary Figures S3D and E. (**D**) The double-floxed and inverted *CD9truc-EGFP* construct are inserted into chromosome 4 of the EVRep mouse, and mice homo- (Tg/Tg) and heterozygous (Tg/WT) can be identified by PCR. The full-length gel is shown in Supplementary Figure S3F.

To obtain cell-specific CD9truc-EGFP expression, we inverted the coding sequence of EV reporter protein CD9truc-EGFP and flanked it by double loxP sites, allowing for CD9truc-EGFP expression driven by a CAG promoter only in Cre recombinase expressing cells (Fig. 2A). HEK293T cells were transiently transfected with CD9truc-EGFP, which only was expressed and yielded green fluorescent cells when co-transfected with Cre recombinase (Fig. 2B). CD9truc-EGFP expression was observed in Cre recombinase co-transfected cells and their corresponding condition medium (Fig. 2C, the full-length blot is shown in Supplemental Figure S3D and E). Next, we used our CD9truc-EGFP plasmid to generate transgenic mice through pro-nuclear injection of the linearized construct. We selected a founder mouse that showed specific and substantial expression of EGFP in Cre-expressing cardiomyocytes when crossed with the tamoxifen-treated *αMHC-MerCreMer* mouse (Supplemental Figure S1B). After backcrossing onto a C57Bl/6 background, we isolated genomic DNA from the liver of a transgene *CD9truc-EGFP* mouse. Nanopore sequencing revealed three reads (approximately 30 kb) containing mouse genomic sequence and CD9truc-EGFP insert sequence. The alignment of the sequenced reads was compatible with an integration of the insert in the mouse genome at approximately chromosomal location 4:99.620.973 (GRCm38.p6). The insert was present in two copies immediately adjacent to each other in opposite directions. The insert does not disrupt any known mouse genes at the location where it is embedded. The area of insertion is, however, an annotated constrained conserved region between multiple eutherian mammals. We designed primers for genotyping (Table 1) that produced PCR products of 449 bp in wild-type mice and 324 bp in homozygotes (Fig. 2D, the full-length gel is shown in Supplemental Figure S3F), enabling us to identify transgenic mice and distinguish heterozygotes from homozygotes.

**Table 1:** Primer sequences for genotyping.

	Sequence	Annealing temperature °C	Function	BP
PS0819	TGCACCTCAA ACACTCAAGC	60	Genotyping of CD9-EGFP forward	449/324
PS0820	TCTTCCTGGA ACACAGCTCA	60	Genotyping of CD9-EGFP reverse 449 bp product in wildtype and heterozygotes	449
PS0821	AGCCAGATTT TTCCTCCTCTC	60	Genotyping of CD9-EGFP 324 bp product in CD9 homo- and heterozygotes (with PS0819)	324

### Tissue-specific EGFP signal in different Cre-positive transgenic mice

**Figure 3:**
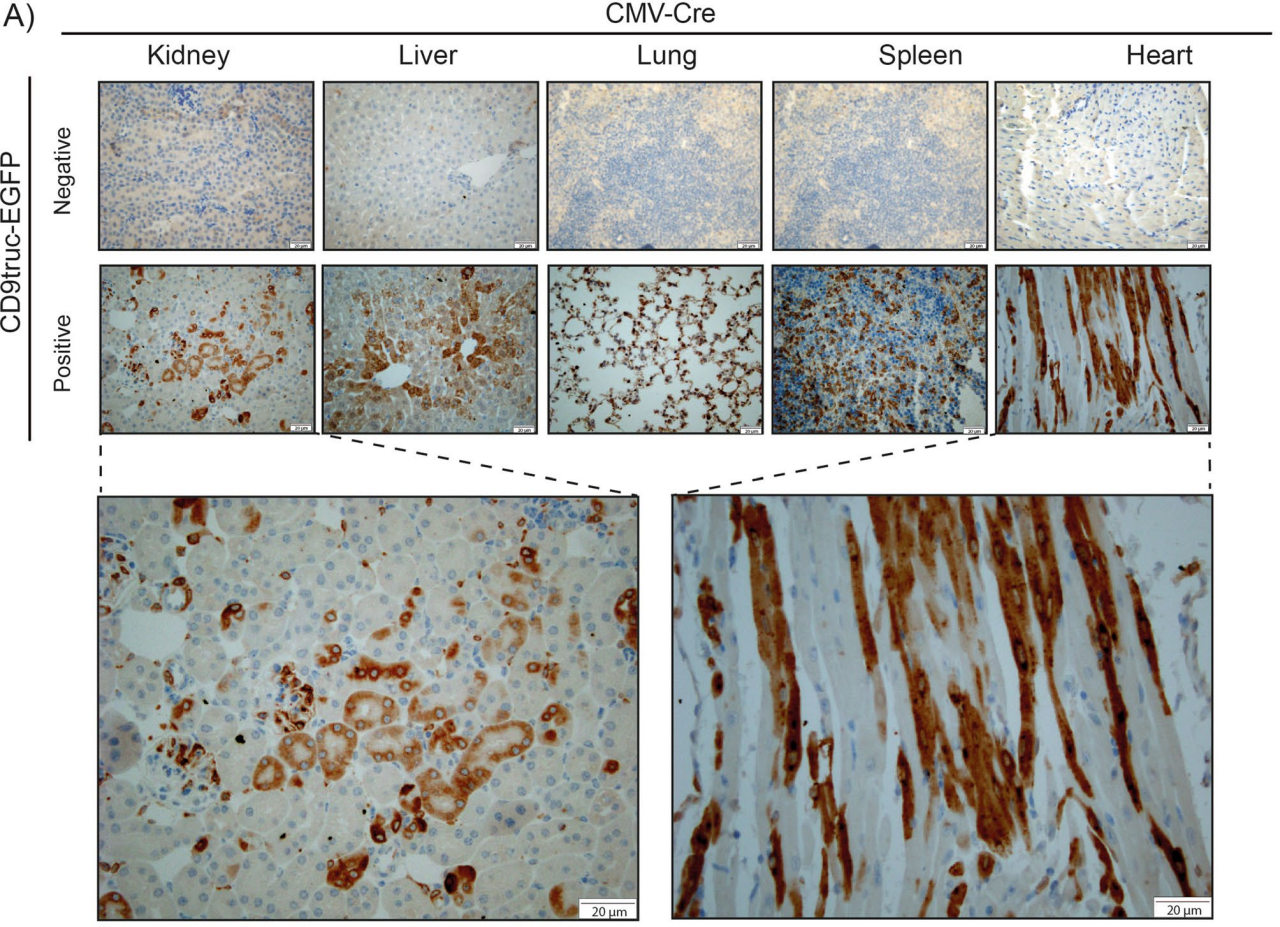
Expression of EGFP in tissue from *CD9truc-EGFP* positive *CD9truc-EGFPxCMV-Cre* mice. (**A**) In contrast to the *CD9truc-EGFP* negative *CD9truc-EGFPxCMV-Cre* mice, EGFP expression is detected by anti-GFP (brown) immunohistochemical staining of paraffin-embedded kidneys, liver, lung, spleens, and hearts of *CD9truc-EGFP* positive *CD9truc-EGFPxCMV-Cre* mice. Scalebar 20 µm. (n = 2).

**Figure 4:**
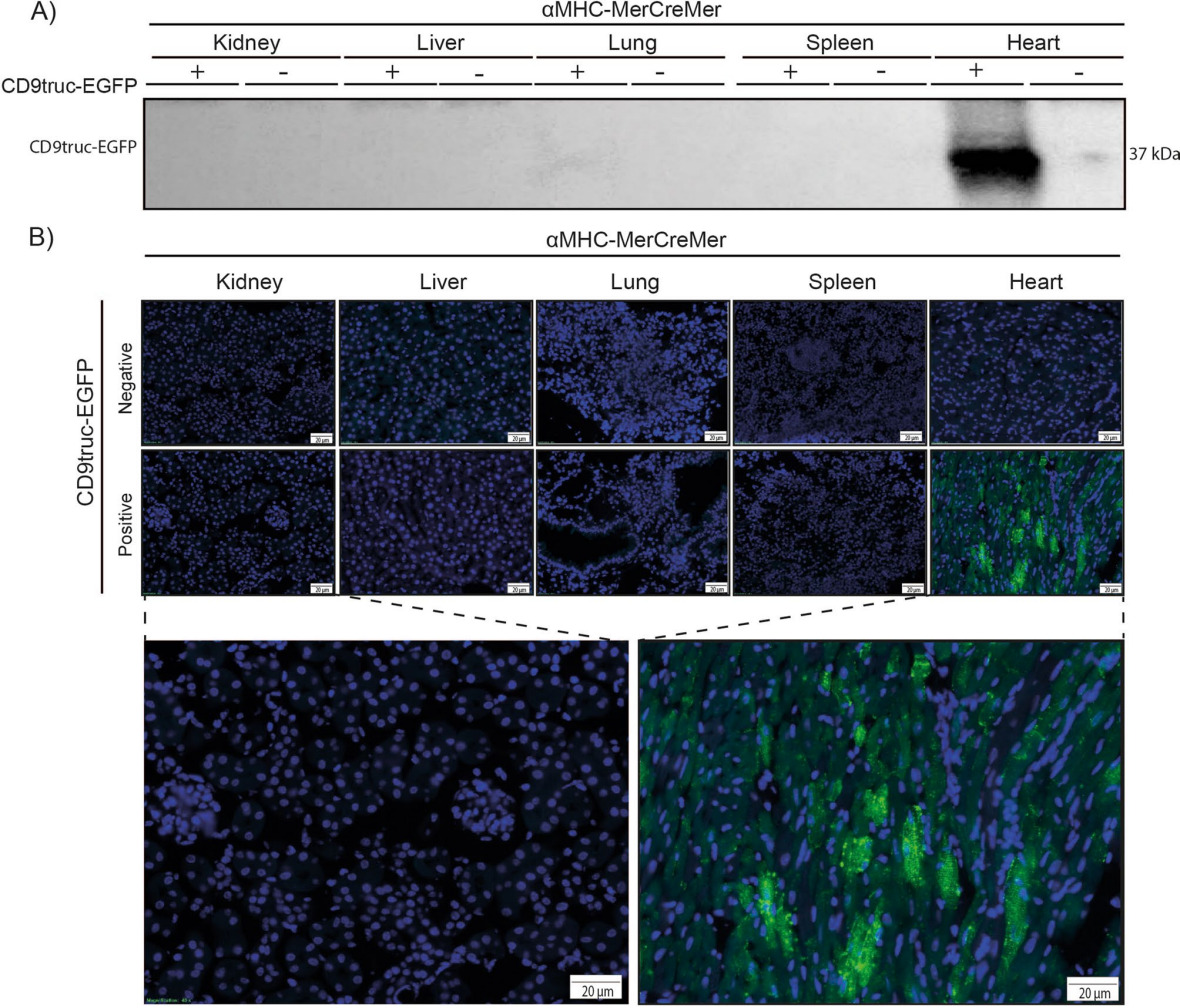
Expression of EGFP in cardiomyocytes from tamoxifen-treated *CD9truc-EGFP* positive *CD9truc-EGFPx*αMHC-MerCreMer mice. (**A**) Tissue homogenates display CD9truc-EGFP expression only in the heart from tamoxifen-treated *CD9truc-EGFP* positive *CD9truc-EGFPxαMHC-Cre* mice. The full-length blot is shown in Supplementary Figure S4A. (**B**) Tissue sections only show EGFP expression (green) in cardiomyocytes from tamoxifen-treated *CD9truc-EGFP* positive *CD9truc-EGFPxαMHC-Cre*. Nuclei (blue). Scalebar 20 µm. (n = 4).

**Figure 5:**
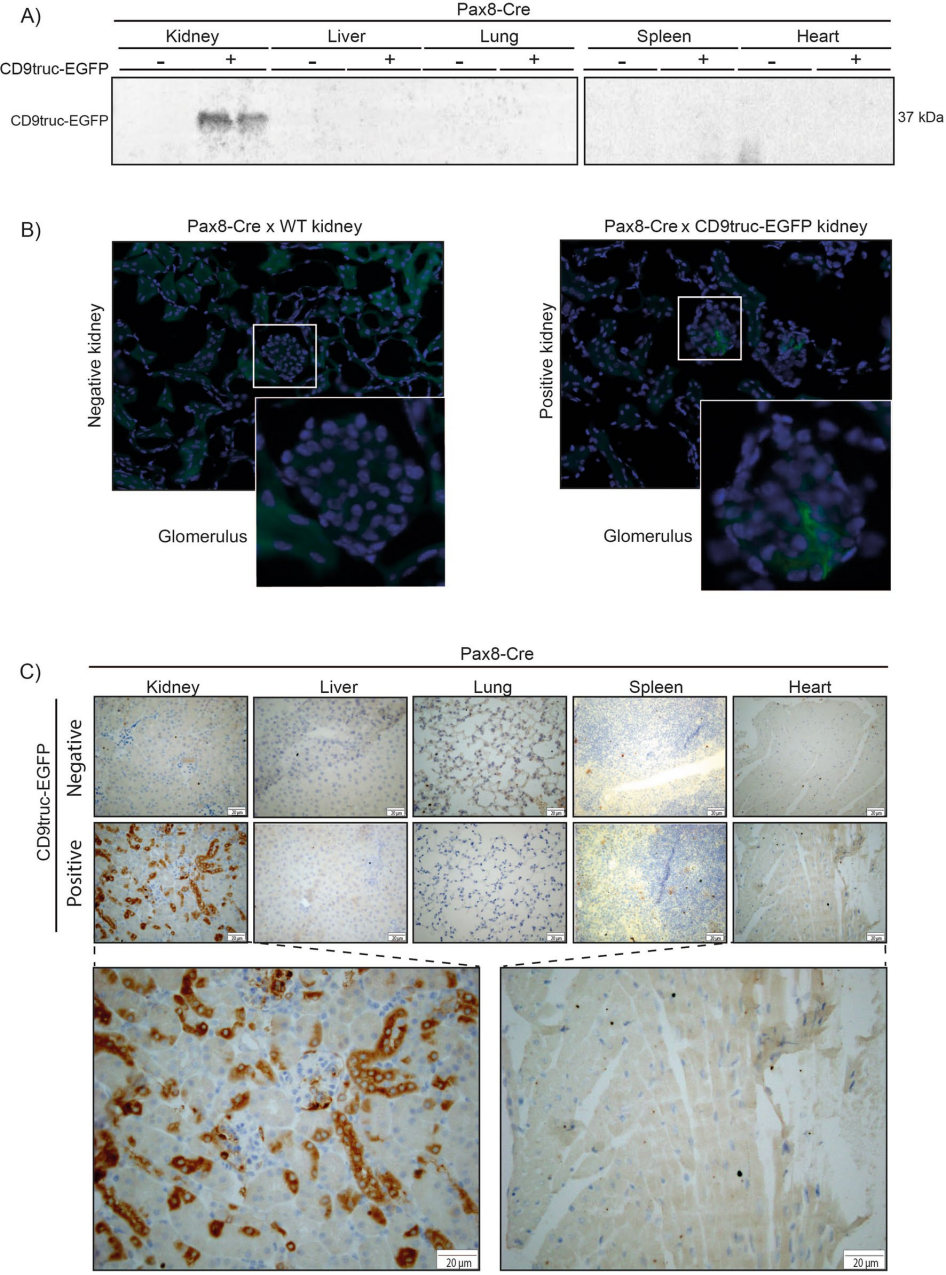
Expression of EGFP in kidney epithelial cells from *CD9truc-EGFP* positive *CD9truc-EGFPxPax8-Cre mice*. (**A**) Tissue homogenates from *CD9truc-EGFP* positive *CD9truc-EGFPxPax8-Cre* mice display CD9truc-EGFP expression in kidneys. Full-length blots are shown in Supplementary Figure S4B. (**B**) Direct fluorescence imaging of kidneys only shows EGFP expression (green) in glomerular cells of *CD9truc-EGFP* positive *CD9truc-EGFPxPax8-Cre* mice. Nuclei (blue). Scalebar 20 µm. (n = 4). (**C**) Immunohistochemical staining of paraffin-embedded kidneys, liver, lung, spleen, and heart from *CD9truc-EGFPxPax8-Cre* mice, EGFP expression is detected by anti-GFP labeling (brown) in epithelial cells in the kidney from only *CD9truc-EGFP* positive *CD9truc-EGFPxPax8-Cre* but not *CD9truc-EGFP* negative littermates. Scalebar 20 µm. (n = 2).

We next used established transgenic Cre recombinase mice to enable tissue-specific activation of the EV reporter protein CD9truc-EGFP. Initially, we crossed the transgene *CD9truc-EGFP* mice with *CMV-Cre* mice^21^ expressing Cre recombinase under the control of the ubiquitously active human cytomegalovirus (CMV) promoter. *CD9truc-EGFP* positive *CD9truc-EGFPxCMV-Cre* mice showed EGFP expression in kidney, liver, lung spleen, and heart by anti-GFP immunohistochemistry (IHC) (Fig. 3A). EGFP was undetectable in any of these tissues in *CD9truc-EGFP* negative littermates (Fig. 3A). To show cell-specific expression of our CD9truc-EGFP construct, we crossed our transgene *CD9truc-EGFP* mice with mice harboring tamoxifen-inducible cardiomyocyte-specific Cre activity (*αMHC-MerCreMer*)^22^ and *Pax8-Cre* mice expressing Cre recombinase kidney tubular epithelium^23^. In *CD9truc-EGFP* positive, tamoxifen-treated *CD9truc-EGFPxαMHC-MerCreMer* mice, EGFP was explicitly detected in the heart by western blotting and direct fluorescence microscopy (Fig. 4A-B, the full-length blot is shown in Supplemental Figure S4A). EGFP was undetectable in kidneys, liver, lung, and spleen in tamoxifen-treated *CD9truc-EGFP* positive and negative *CD9truc-EGFPxαMHC-Cre* mice and the hearts of tamoxifen-treated *CD9truc-*EGFP negative *CD9truc-EGFPxαMHC-Cre* littermates (Fig. 4A). Likewise, we found EGFP expression specifically in the kidney of *CD9truc-EGFP* positive *CD9truc-EGFPxPax8-Cre* mice using western blotting (Fig. 5A, the full-length blot is shown in Supplemental Figure S4B). By fluorescence microscopy, however, green fluorescence was not directly detectable in the kidney epithelium of kidney cryo-sections, and only low EGFP signals were associated with glomeruli (Fig. 5B). The preparation of cryo-sections involved exposure of the tissue to large osmotic gradients, and we tested whether epithelial EGFP expression was present in paraffin-embedded formalin-fixed kidneys. Using an anti-EGFP antibody, we detected kidney-specific EGFP expression in *CD9truc-EGFP* positive but not negative, *CD9truc-EGFPxPax8-Cre* mice (Fig. 5C). Thus, these data demonstrate that our transgene *CD9truc-EGFP* mouse, which we term EVRep, allows for cell-specific expression of CD9truc-EGFP.

### Isolation of EGFP-positive extracellular vesicles from plasma and urine

**Figure 6:**
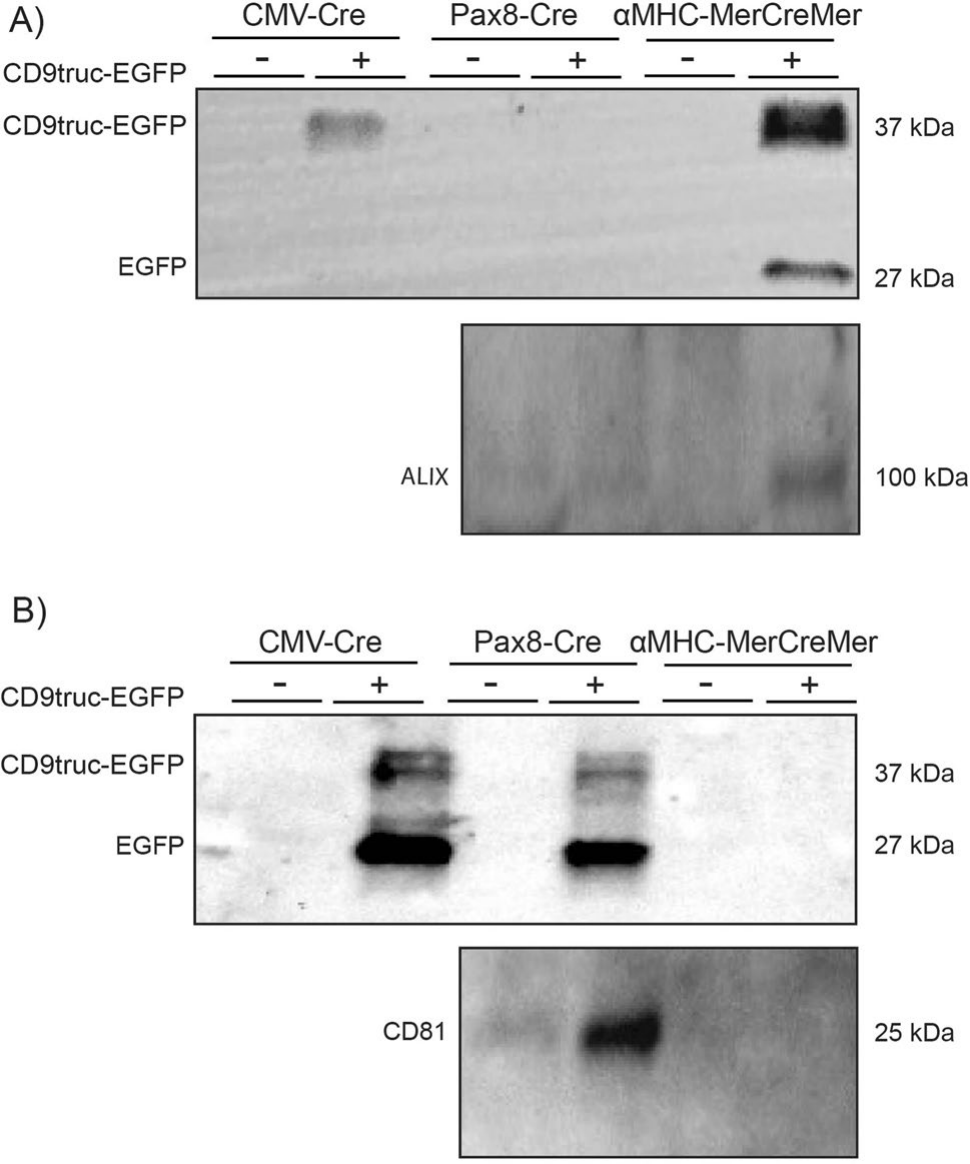
Isolation of EVs from plasma and urine by anti-GFP precipitation of plasma and urine samples from *CD9truc-EGFP* transgene mice. (**A**) CD9truc-EGFP was only isolated from *CD9truc-EGFP* positive *CD9truc-EGFPxCMV-Cre* and tamoxifen-treated *CD9truc-EGFPxαMHC* but not *CD9truc-EGFPxPax8-Cre* mice or *CD9truc-EGFP* negative littermates. The EV marker ALIX was co-isolated with CD9truc-EGFP in plasma samples from *CD9truc-EGFP* positive *CD9truc-EGFPxαMHC-Cre* only. Full-length blots are shown in Supplementary Figure S5A and B. (**B**) CD9truc-EGFP was only detected by anti-GFP immunoprecipitation of urine samples from *CD9truc-EGFP* positive *CD9truc-EGFPxCMV-Cre* and *CD9truc-EGFPx*Pax8-Cre mice, but not tamoxifen-treated *CD9truc-EGFPxαMHC-*Cre or *CD9truc-EGFP* negative littermates. The EV marker CD81 co-precipitated with CD9truc-EGFP only in urine samples from *CD9truc-EGFP* positive *CD9truc-EGFPxPax8-Cre* mice. Full-length blots are shown in Supplementary Figures S5C and D.

Since EVs have been detected in most body fluids, we next used plasma and urine samples from *CD9truc-EGFP* positive and negative *CD9truc-EGFPxCMV-Cre*, tamoxifen-treated *CD9truc-EGFPxαMHC-MerCreMer*, and *CD9truc-EGFPxPax8-Cre* mice to determine the cell-specific contribution of EVs to plasma and urine fluid compartments. EVs were precipitated and isolated from plasma using anti-GFP nanobody conjugated beads. CD9truc-EGFP was detected in plasma from *CD9truc-EGFP* positive *CD9truc-EGFPxCMV-Cre* and tamoxifen-treated *CD9truc-EGFPxαMHC-Cre* mice, but not from *CD9truc-EGFPxPax8-Cre* and *CD9truc-EGFP* negative littermates (Fig. 6A, the full-length blot is shown in Supplemental Figure S5A). The EV marker ALIX co-precipitated with CD9truc-EGFP only in plasma from tamoxifen-treated *CD9truc-*EGFP positive *CD9truc-EGFPxαMHC-Cre* mice (Fig. 6A, the full-length blot is shown in Supplemental Figure S5B). In urine samples, CD9truc-EGFP was observed in *CD9truc-EGFP* positive *CD9truc-EGFPxCMV-Cre,* and *CD9truc-EGFPxPax8-Cre* mice, whereas tamoxifen-treated *CD9truc-EGFPxαMHC-Cre* urine samples were devoid of detectable CD9truc-EGFP (Fig. 6B, the full-length blot is shown in Supplemental Figure S5C). The EV marker CD81 co-precipitated with CD9truc-EGFP only in urine samples from *CD9truc-EGFP positive CD9truc-EGFP*x*Pax8-Cre* (Fig. 6B, the full-length blot is shown in Supplemental Figure S5D). Together, these data suggest that the EVRep mouse allows for the isolation of cell-specific EVs from biological fluids.

## Discussion

Using a genetically encoded CD9truc-EGFP fusion protein that displays EGFP on EVs combined with a Cre-dependent switch, we have created a transgenic mouse enabling EV detection at the cellular level as well as rapid and selective isolation of cell-specific EVs from plasma and urine. This EVRep mouse model is thus the first genetic EV reporter model that allows easy tracking and isolation of EVs in vivo in mice. Therefore, we believe that this novel tool can be utilized to generate novel insights into EV biology.

Cell-to-cell EV communication has already been demonstrated in several settings. In vitro, the transport of miRNAs in EVs act as a neuron-to-astrocyte communication pathway in the central nervous system^24^, and stem cell-derived EVs appear to target bone marrow and peripheral sites^25^. In vivo, intravascular administration of mesenchymal stem cell-derived EVs (MSC-EV) labeled with PKH26 dye closely resembles the positive effect of MSCs on postischemic recovery after acute tubular injury^26^. Another example includes intravenous injection of endothelial progenitor cell-derived EVs labeled with PKH26 dye isolated from cell-conditioned medium that protects from complement-mediated mesangial injury after experimental anti-Thy1.1 glomerulonephritis^27^. Furthermore, similar positive effects have been obtained in the myocardium where intravenous administration of EVs isolated from plasma protected against Ischemia–Reperfusion Injury^28^. Altogether, this suggests that EVs retain regenerative abilities. However, for most EV studies, such injections cause a supra-physiological concentration of EVs from a single cell type, and the naturally occurring in vivo concentrations might not be sufficient to cause similar effects. Furthermore, important paracrine and autocrine effects might be altered or unobserved with this approach and affect the in vivo distribution and the biological function of the EVs. Importantly, injected EVs often accumulate in the liver, spleen, and lungs regardless of the origin^26,27,29^. We did not observe CD9truc-EGFP accumulation in these organs under normal physiological circumstances. These observations emphasize some of the hurdles with injections of labeled EVs^30,31^, which may be overcome using the EVRep mouse developed to enable a robust EV fate mapping.

EVs isolated from biological fluid provide access to cell-derived lipids, RNAs, and proteins; however, the representation of EVs from different cell types in, for example, urine and plasma is not well described. Consistent with a previous study^32^, we found that EVs from cardiomyocytes were readily detectable in plasma samples. Intravascular injection of labeled EVs isolated from serum have been detected in urine^33^; however, we did not detect cardiomyocyte-derived EVs in urine, indicating that EVs are not freely filtered across the glomerular filtration barrier in the kidney, probably as a result of their size and negative charge^34–36^. On the other hand, we detected kidney epithelial EVs in urine. This is consistent with our previous findings using proteomic database analysis showing that 99.96% of urinary EV-associated proteins are likely to originate from the kidney, the urinary tract epithelium, and the male reproductive tract in humans^37^. It should be noted that, for example, the *CD9truc-EGFPxPax8-Cre* mice only showed CD9truc-EGFP in some of the tubular epithelial cells; thus, methods with higher sensitivity than western blotting may be able to detect cardiomyocyte and renal tubular epithelial-derived EVs in plasma and urine. Nonetheless, the distribution of EVs appears to be fluid compartment restricted, and EVs isolated from different fluid compartments, e.g., plasma versus urine, may only represent a subset of cell types in the body.

We found that kidney epithelial-derived EV signal was significantly affected by preparation of frozen kidney cross-sections before fluorescence microscopy, while cardiomyocyte EV tissue abundance was less affected. Western blotting suggested that CD9truc-EGFP was abundantly expressed in *CD9truc-*EGFP positive *CD9truc-EGFPxPax8-Cre* mice, but in frozen kidney sections, the EGFP was only slightly visible in the glomerulus of kidneys by directed fluorescence microscopy. The reason for this discrepancy is not known, but renal epithelial cells are, however, highly water permeable^38^, and we speculate that rapid osmotic changes (e.g., 25% sucrose) imposed during tissue preparation are involved. Recently, it was shown that the handling and preparation of tissue samples significantly affect tissue EV abundance through release to, e.g., washing buffers^39^. Our observations agree with this and suggest that careful and specific tissue preparation is essential when analyzing tissue EV levels and intercellular communication.

Similar to our approach, three other studies have used genetic labeling of EVs^15,18,40^ to track the faith of endogenous EVs. While their use of an intravesicular localization of the reporter proteins prevents affinity isolation, our approach shares the limitation that there is a risk that only a subpopulation of EVs is labeled. Notably, the number of identified EV subtypes is continuously growing, and there is no consensus on which protein markers represent the different populations. Thus, we cannot exclude that genetic labeling using CD9 may add a bias in the downstream analysis, as it may only represent EV fractions. Moreover, we used a truncated version of CD9 in which the large extracellular loop was deleted. The large extracellular loop mediates many lateral CD9 interactions, and its deletion might reduce potential adverse effects of its overexpression; however, the CD9truc-EGFP may not show the exact same behavior as wild-type CD9. Nonetheless, the ease at which cell-specific EVs can be isolated using the EVRep mouse will benefit a more comprehensive characterization and identification of EV subtypes and enable other cell-specific EV markers to be identified.

In summary, our novel transgenic EVRep mouse allows easy in vivo tracking and isolation of EVs and can be used to elucidate EV biology and their further use.

## Methods

### EV track data

We have submitted all relevant data of our experiments to the EV-TRACK knowledgebase (EV-TRACK ID: EV210297)^41^.

### Plasmids

The genes encoding truncated CD9 fused to EGFP, and the doubled-floxed and inverted version were synthesized and cloned into plasmid pcDNA3.1 by BioCat GMBH. The coding sequence is shown in Supplemental Figure S1C. The double-floxed, inverted CD9truc-EGFP from the pcDNA3.1 plasmid was amplified by PCR and ligated into pCAG-Cre (a gift from Connie Cepko; Addgene plasmid # 13775) digested with EcoRI and NotI (New England Biolabs). All constructs were verified by Sanger sequencing at Eurofins Genomics. mRFP-Rab5 was a gift from Ari Helenius (Addgene plasmid # 14437; http://n2t.net/addgene:14437; RRID:Addgene_14437), mCherry-ER (Addgene plasmid # 55041; http://n2t.net/addgene:55041; RRID:Addgene_55041) and mTagBFP2-CD81-10 (Addgene plasmid # 55281; http://n2t.net/addgene:55281; RRID:Addgene_55281) were gifts from Michael Davidson.

### Cell cultures

Mouse epithelial M1 (ATCC, Virginia, United States) cells were cultured as previously described^42^. For analysis of cell-conditioned medium, PC1 serum-free medium (Lonza, No. 344018, Walkersville, MD, USA) was used. The M1 cells with stable CD9truc-EGFP expression were generated by transfection with pcDNA3.1 CD9truc-EGFP using Metafectene Pro (Biontex, München, Germany) and selection with G418 (InvivoGen). HEK293T (ATCC, Virginia, United States) cells were maintained in Dulbecco's Modified Eagle Medium, F-12 Nutrient Mixture (Gibco, Sigma Aldrich, Denmark) containing 10% fetal bovine serum (FBS, Fisher Scientific, DK) and 1% Penicillin–Streptomycin in a 5% CO2 humidified incubator at 37 °C. Harvesting of cells and medium were performed when cells reached 80% confluence in 25 cm^2^-flasks.

### Animal models

We linearized pCAG-DIO-CD9truc-EGFP with HindIII and SalI (New England Biolabs) and gel extracted (New England Biolabs) the 3649-bp fragment containing the transgene. The purified fragment was used to generate transgenic mice with genomic integration of the CAG-promotor driven double-floxed inverted CD9truc-EGFP by pro-nucleus injection^43^. The *αMHC-MerCreMer* strain (JAX stock #005650, The Jackson Laboratory, Bar Harbor, ME, USA) expresses inducible Cre in cardiomyocytes upon tamoxifen treatment, and it is maintained by homozygous breeding^22^. The *CMV-Cre* (JAX stock #006054), The Jackson Laboratory, Bar Harbor, ME, USA) expresses Cre in all tissues and is maintained by homozygous breeding^21^. The B6.129P2(Cg)-Pax8^tm1.1(cre)Mbu^/J (JAX stock #028196), The Jackson Laboratory, Bar Harbor, ME, USA) strain referred to as Pax8-Cre expresses Cre recombinase in known Pax8 expression domains including the developing thyroid gland, inner ear, kidney, and mid-hindbrain region^23^. The animal experiment was performed in accordance with Danish Law and approved by the Danish Animal Experimentation Council (#2019-15-0201-01644 and 2019-15-0202-00052). The study was performed in accordance with ARRIVE guidelines.

### Sample collection

7–9 weeks old mice were transferred individually to metabolic cages (12:12 h light–dark cycle, 28 ± 1 °C) for three days with free access to water and regular rodent diet (LabDiet® 5001, Forth Worth, TX, USA). After 24 h acclimation, urine samples were collected on days 2 and 3. Subsequently, mice were used for perfusion fixation or direct organ harvest, as described below. Urine was stored at − 80 °C and a protease inhibitor (1:1000, P8340, Protease Inhibitor Cocktail, Sigma Aldrich, Denmark) were added when thawed. Mice used for immunohistochemistry and fluorescent microscopy were anesthetized by i.p. injection with 10 mg/kg Xylazine (Rompun, Bayer Healthcare, Shawnee Mission, KS) and 50 mg/kg Ketamine (Ketalar, Pfizer, Sandwich, Kent, UK). Blood samples were taken by cardiac puncture through the apex before mice were flushed with 1 × PBS and fixed with 4% paraformaldehyde by retrograde perfusion via the left ventricle. Subsequently, mice were fixed for an additional 6 h in paraformaldehyde and transferred to 1 × PBS. Mice used for western blotting were likewise anesthetized, blood samples were taken, and relevant organs were removed and snap-frozen in liquid nitrogen.

### Immunohistochemistry and fluorescence microscopy

Staining was performed on 2 μm paraffin-embedded tissue samples deparaffined in xylene and rehydrated in decreasing ethanol solutions (99–70%) followed by target retrieval in heated TEG buffer (1 mmol/L Tris, 0.5 mM EGTA, pH 9.0). After cooling, slides were exposed to a 50 mM NH_4_Cl and 0.3% H_2_O_2_ solution for 10 min, washed in 1 × PBS, and incubated in 1 × PBS with 0.3% Triton for 30 min at room temperature. Next, the slides were incubated with primary antibody (Anti-GFP) diluted in 1 × PBS with 0.3% Triton × 100 at 4 °C overnight.

Next, slides were incubated for 30 min at room temperature, washed in 1 × PBS + 0.05% tween five times, and incubated with secondary HRP antibody in PBS + 0.05% tween for 1 h at room temperature. Slides were then washed in PBS, and HRP was visualized by DAB-staining (3,3’-diaminobenzidine). Lastly, slides were stained in hematoxylin mounted with Aquatex (Merck KGaA, Darmstadt, Germany).

For fluorescence microscopy, snap-frozen organs were removed from mice and placed in 1 × PBS with 0.05% acid and 25% sucrose overnight. Next, organs were placed in 1 × PBS with 0.05% acid and 50% sucrose for 2 h, embedded in OCT tissue freezing medium and 5 μm cross-sectioned on a Cryostat (Leica CM3050 S, Leica Biosystems, USA). Cross-sections were placed in 1 × TBS for 10 min, dried, and one drop of Slowfade™ Gold antifade reagent with DAPI (Invitrogen, Thermo Fisher Scientific, Eugene, OR USA) was added before mounting. All tissue and cell samples were obtained using an Olympus BX51 Fluorescence microscope.

### Isolation of extracellular vesicles from cells and medium

48 h before, the cell medium was changed to PC1 serum-free medium (Lonza, No. 344018, Walkersville, MD, USA). Cells were lysed in RIPA Lysis buffer for 1 h at 4 °C on an orbital shaker. Next, 25 cm cell scrapers were used to release cells from the flask into the RIPA buffer. The solution was then centrifugated at 13,000*g* at 4 °C for 10 min, and the supernatant was stored in new tubes at − 80 °C. The medium was transferred to 15 mL tubes and centrifugated for 10 min at 5000 g. The supernatant was then transferred to new tubes and stored at − 80 °C. To isolate EVs, the thawed medium was centrifugated for 15 min at 5000 g at 4 °C. The supernatant was mixed with an equal amount of freshly made ExtraPEG^44^ (16% PEG-6000, Sigma Aldrich, 1 M NaCl, Milli-Q water) and left in a Multi-Rotator at 4 °C overnight. Next, samples were centrifugated for 15 min at 5000 g at 4 °C, and the supernatant was discarded. The remaining pellet was resuspended in 100 μl × 1 RIPA buffer and stored at − 20 °C until further use.

### Immunofluorescence of cells

Cells were seeded on coverslips in multiple 12-wells plates (Biocoat Cell environments, Poly-D-Lysine Cellware) and given 24 h to attach to the coverslips. In the experiments with transfection of fluorescent organelle markers, the cells were transfected the day after seeding on coverslips using Metafectene Pro (Biontex, München, Germany) and grown for 24 h. Next, cells were fixed in 4% paraformaldehyde for 10 min and rinsed 2 times in 1 × PBS with 1 mM MgCl_2_ and 0.1 mM CaCl_2_, followed by permeabilization with 0.3% Triton in 1 × PBS for 15 min and another wash in 1 × PBS with 1 mM MgCl_2_ and 0.1 mM CaCl_2_. Cells where then incubated in 300 μl primary antibody (Table 2) at 4 °C overnight washed 3 times in 1xPBS and incubated with 300 μl secondary antibody for 1 h at room temperature. Afterward, cells were incubated with 4’,6-diamidino-2-phenylindole (D9542-10MG DAPI, Sigma Aldrich, Denmark) to stain DNA followed by 5 washes in 1 × PBS. Lastly, coverslips containing cells were mounted with fluorescent mounting media (DAKO, Carpinteria, CA, USA) on coverslips. ImageJ, version 2.0.0-RC-43/1.10e) was used to analyze pictures.

**Table 2:** Primary and secondary antibodies.

Target protein	Company	Catalog #	Dilution
**Immunocytochemistry**			
**Primary antibody**			
Anti-hCD9	RD systems	MAB1880	1:50
**Secondary antibody**			
Anti-mouse AlexaFlour 568	Abcam, UK		1:500
**Western blot**			
**Primary antibodies**			
GFP	Nordic BioSite, SE	GTX113617	1:1000
ALIX	Cell Signaling Technology, NL	2171	1:1000
Flotilin	Abcam, UK	Ab13493	1:1000
ß-Actin	Abcam, UK	Ab8227	1:10,000
CD81	Santa Cruz Biotechnology, USA	E1817	1:1000
TSG-101	Abcam, UK	Ab30871	1:1000
Immunohistochemistry	**Primary antibodies**		
Anti-GFP	Abcam, UK	Ab6673	1:200
**Sencondary antibodies**			
Polyclonal Goat Anti-Mouse immunoglobulins/HRP		DAKO, DK	P0447
Polyclonal Goat Anti-Rabbit immunoglobulins/HRP		DAKO, DK	P0448
Polyclonal Rabbit Anti-Goat immunoglobulins/HRP		DAKO, DK	P0449

### Immunoblotting

Samples were mixed with LDS sample buffer (NuPAGE, Invitrogen, Thermo Fischer Scientific, Van Allen Way, Carlsbad, USA) and sample reducing agent (NuPAGE, Invitrogen, Thermo Fischer Scientific, Van Allen Way, Carlsbad, USA) and heated for 10 min before they ran on a gel. For cell and tissue fractions, 10 μg was loaded per well. The protein was then transferred to a membrane activated in 99% Ethanol. Afterward, the membrane was blocked for 30 min in 5% skimmed milk and incubated overnight with primary antibodies at 4 °C (Table 2). Following, membranes were washed 3 times in Tris-Buffered saline with Tween-20 (TBST, 20 mM Tris-base, 137 nM NaCl, 0.05% Tween-20 (Merck), pH 7.6) and incubated with secondary antibody (Table 2) for 1 h at room temperature. Next, membranes were washed 3 times in TBS-T, and proteins were visualized by ECL plus a Molecular Imager (ChemiDoc XRS + , BIO-RAD) with Image Lab software (BIO-RAD).

### Immunoprecipitation of EGFP associated EVs

EVs containing EGFP were precipitated from 200 μl plasma and 750 μl urine with extraPEG as described above, except that the pellet was resuspended in 100 μl 1 × PBS to keep EVs intact. EVs immunoprecipitated using ChromoTek GFP-trap Magnetic Beads (Chromotek GmbH, Germany). 25 μl beads were added to a 1.5 ml tube and rinsed with 1000 μl ice-cold 1 × PBS. Next, plasma or urine was added to the equilibrated beads, and tubes were placed in a Multi-Rotator (Grant-bio, PTR-35) at 4 °C for 1 h. Samples were when placed back in the magnet and allowed to attach before the supernatant was discarded. Subsequently, beads were resuspended in 1 mL ice-cold 1 × PBS-T, and the samples were placed back in the magnet. The supernatant was discarded after 3 min when the beads were attached to the magnet, and this step was repeated 4 times.

### Tunable resistive pulse sensing

A qNano platform with a Nanopore NP150 (Izon Science, Oxford, UK) and polystyrene calibration beads CPC200 (Izon Science), Oxford, UK) were used for calibration of relative particle size and speed. 35 μl cell media was loaded, and analyses were performed according to manufactory instructions.

### Size exclusion chromatography

A qEVoriginal/35 nm SMART column (Izon Science, Oxford, UK) with optimal separation size between 35–350 nm was used to isolate EVs from 0.5 ml from M1-CD9-EGFP medium according to manufacturer's instructions. 0.5 mL fractions were collected, and western blotting was used to verify the presence of EGFP. To verify that proteins mainly were eluted in the last fractions, proteins concentrations were measured in each fraction with DC Protein Assay (Biorad, California, U. S.) according to the manufacturer's guidelines.

### Nanopore sequencing

Mouse liver from a *CD9truc-EGFP* positive EVRep mouse was homogenized, and DNA was extracted using the Nanobind Tissue Big DNA Kit (Circulomics, Baltimore, MD, USA) according to the manufacturer's instructions. The DNA was prepared for Oxford Nanopore sequencing using the Ligation Sequencing Kit (Oxford Nanopore Technologies, Oxford, UK). The prepared library was then sequenced using one flow cell on the Oxford Nanopore PromethION sequencing platform. Base-calling was performed with MinKNOW software, and FASTQ files were aligned to a custom mouse genome (GRCm38.p6) containing the *CD9truc-EGFP* insert and accessory sequences as an extra chromosome. Alignment was performed using Minimap2^45^. Data were visualized using IGV ver2.8.1 (Broad Institute), Genome Ribbon^46^, and NCBI BLAST.

## Supplementary Information

Supplementary Information 1.

Supplementary Information 2.

Supplementary Information 3.
