# Supplementary material for: A new transgene mouse model using an extravesicular EGFP tag enables affinity isolation of cell-specific extracellular vesicles
Source: Sci Rep. 2022 Jan 11;12:496. doi: 10.1038/s41598-021-04512-0 (PMC8752749; doi:10.1038/s41598-021-04512-0)
Supplement: Supplementary file 3 — Supplementary Information 3. [file 41598_2021_4512_MOESM3_ESM.docx]

**Supplementary Figure Legends**
